# Does Personality, Trait Emotion Regulation, and Trait Attentional Control Contribute toward the Experience and Impact of an Alcohol Hangover?

**DOI:** 10.3390/healthcare11071033

**Published:** 2023-04-04

**Authors:** Felicity Hudson, Craig Gunn

**Affiliations:** School of Psychological Science, University of Bristol, Bristol BS8 1TQ, UK

**Keywords:** alcohol, hangover, personality, emotion regulation, attentional control

## Abstract

Mixed results have been reported for the relationship between personality and hangover, but recent findings have indicated that regulatory and attentional control processes may relate to hangover severity and the impact of a hangover on completing daily activities. This study aimed to explore how these factors relate to hangover severity, hangover impact, and to unhealthy alcohol use. In total, 108 participants completed a survey, rating the severity and impact of their last-experienced hangover and completing measures of the above factors. Separate multiple linear regressions were conducted to analyse each outcome (severity, impact, unhealthy drinking). For severity, the overall regression was significant (Adj. R^2^ = 0.46, *p* < 0.001), with the attentional control factor ‘Focusing’ (*B* = −0.096, *p* = 0.011), and personality factor ‘Agreeableness’ (*B* = 0.072, *p* = 0.005) predicting severity. For impact, the overall regression was significant (Adj. R^2^ = 0.41, *p* < 0.001) with the attentional control factor ‘Shifting’ (*B* = −0.252, *p* = 0.021), personality factors ‘Extraversion’ (*B* = 0.225, *p* = 0.009) and ‘Agreeableness’ (*B* = −0.156, *p* = 0.042), and hangover severity (*B* = 1.603, *p* < 0.001) predicting impact. For unhealthy drinking, the overall regression model was significant (Adj. R^2^ = 0.45, *p* < 0.001) with emotion dysregulation factors ‘Awareness’ (*B* = 0.301, *p* = 0.044) and ‘Impulse Control’ (*B* = 0.381, *p* = 0.011) predicting unhealthy drinking. These findings add to our understanding of the heterogeneity of hangover experience and highlight that attentional control, emotion regulation, and personality play important roles in the experience and impact of a hangover.

## 1. Introduction

Alcohol hangover is the most common negative consequence of heavy alcohol consumption [1]. It is defined as a combination of negative mental and physical symptoms (e.g., headache, confusion) following an episode of alcohol consumption, occurring when Blood Alcohol Concentration is approaching zero [2]. Hangover can have a wide-ranging impact on cognitive [3] and emotional processes [4], which could in turn affect day-to-day activities. Although there is growing understanding of the physiological factors that contribute toward a hangover [5], relatively little is known about the psychological factors involved. Participants often report a wide range of symptoms experienced at varying intensity, and the current study aims to explore how personality, emotion regulation, and attentional control may influence the experience and impact of a hangover, as well as the links to unhealthy alcohol use.

Few studies have explored how personality could relate to overall hangover severity. One early study by Harburg et al. [6] suggested that neuroticism may predict hangover, but they have since been criticized for including participants who reported no hangover symptoms. Recently, Terpstra et al. [7] re-examined the link between personality and overall severity with results suggesting no association with any of the big five personality factors (Extraversion, Agreeableness, Conscientiousness, Neuroticism, Openness). These results are surprising given that individuals who score high on extraversion are more likely to binge drink [8] and greater frequency of hangovers is associated with greater subjective severity [9]. Furthermore, the way a person is able to cope with an adverse event has been linked with severity. Avoidant coping styles [7], i.e., those whereby individuals deny, minimize or otherwise avoid dealing with emotional turmoil, and emotion dysregulation [10] are both positively associated with hangover severity. In addition, qualitative research suggests that individuals may be better able to cope with hangover experiences through communal bonding, where they discuss the previous night’s activities and how they are currently feeling [11]. Personalities such as those high in optimism, extraversion, conscientiousness, and openness are linked to higher levels of engagement coping, which involves seeking social support and actively confronting adverse situations [12]. On the other hand, neuroticism is linked to disengagement or avoidant coping. It is therefore possible that personality is linked to the severity and impact of a hangover through the strategies that individuals adopt to cope with their hangover symptoms. 

Alcohol hangover can have impairing cognitive effects, both in terms of ‘core’ cognitive functions such as memory and psychomotor skills [3], as well as higher-order executive functions [13,14]. One explanation is that alcohol hangover reduces available attentional resources that can be utilized to complete complex tasks [15]. Participants also report needing to exert greater effort when completing tasks during a hangover relative to a no-hangover control, which further suggests a reduction in available attentional resources [13,16]. This available resource reduction may occur due to attention-consuming symptoms (e.g., headache; [17], poorer information processing [18] or both. Exploring the link between attentional control and symptoms could improve our understanding about the nature of this relationship. As attentional control is used when overcoming consuming stimuli and hangover impairs cognitive processes such as multi-tasking [19], it may also be associated with the ability to complete daily activities whilst hungover.

Being able to cope with negative experiences relies on an individual’s ability to regulate emotions. Previous research has found individuals are able to effectively regulate during a hangover through the exertion of additional effort, but experience a general negative shift in the appraisal of stimuli relative to a no-hangover control [13]. Furthermore, state-based scores in subjective difficulties for regulating emotions are greater when hungover than not, and scores are related to current hangover severity. As previously mentioned, recent survey data have also indicated that avoidant coping is positively related to severity, suggesting that individuals with maladapted coping strategies may experience more severe hangovers [7]. Together, these data suggest that our inherent abilities to cope with difficulty relate to how hangover is experienced, and that we perceive greater difficulties regulating emotions whilst hungover. 

Another aspect of coping with a hangover is the impact it has on our ability to perform daily activities, such as household chores. Individuals express frustration at forsaking daily tasks to overcome aversive hangover symptoms [11,20], yet little research has explored factors that influence the ability to complete daily activities during a hangover. Furthermore, hangover has tentatively been linked to future problem drinking behaviours [21], and our emotional response during a hangover, such as increases in feelings of anxiety, have been linked to unhealthy drinking [22]. Personalities such as extraversion and emotion regulation have been linked to an increased likelihood of developing an alcohol use disorder [8,23,24]. In addition, attentional control may moderate the relationship between cognitive biases toward alcohol and alcohol use [25]. Therefore, this study will also explore whether each of these factors underlie the tentative link between hangover and unhealthy drinking. Personality factors were explored for their link with hangover severity, and it was hypothesised that extraversion would predict hangover severity, a lower impact of a hangover, and more unhealthy drinking. It was also hypothesised that attentional control would predict lower hangover severity and hangover impact, whilst difficulties in regulating emotion would predict greater severity, impact, and unhealthy drinking behaviours.

## 2. Materials and Methods

### 2.1. Participants

In total, 198 participants were recruited. Partial responses (*n* = 88) and responses that were clear outliers or were of uncertain data quality (e.g., reporting 1000 units of alcohol consumed in a single session; *n* = 2) were removed. The remaining 108 participants (30 male, 78 female), aged 18–59 (*M* = 23.22, *SD* = 7.58), completed the study. Participants self-reported good mental and physical health, regularly drank 6 (female) or 8 (male) units of alcohol on one occasion, and had experienced a hangover in the past month. Participants were excluded if they were pregnant or breastfeeding, on any medication that interacts with alcohol, or had any personal or family history of alcohol or drug dependency. The study was approved by the Research Ethics Committee at the University of Bristol (code: 10909).

### 2.2. Design

Participants completed the study’s online survey through Qualtrics. Questions assessed previous hangover severity, personality, attentional control, and difficulties in emotion regulation.

### 2.3. Measures

Participants were asked about their drinking habits and the amount of alcohol consumed prior to the last hangover they experienced. Alcohol hangover was assessed using the modified Alcohol Hangover Severity Scale (mAHSS) [26]. This composite measure asks participants to rate how severely they experienced a list of 22 symptoms during their last hangover on an 11-point scale (0 = absent, 10 = extremely severe).

The Impact of Illnesses Scale (IIS) is a brief 9-item questionnaire assessing the impact an illness has had on day-to-day functioning [27]. Participants are asked to rate the extent that their ‘illness’ impacts upon several statements reflecting common day-to-day tasks (e.g., enjoyable recreational activities, or routine chores) using a 4-point Likert scale (0 = Not at all, 3 = Fully). In the current study, ‘illness’ was adapted to say ‘hangover’ and responses indicated high internal consistency (α = 0.88). 

The Alcohol Use Disorder Identification Toolkit (AUDIT) was used to measure unhealthy alcohol use [28]. The 10-item questionnaire asks participants about their alcohol intake, potential dependence on alcohol, and their experience of alcohol-related harm. Each item has a possible score of 0–4, with the exception of items 9 and 10, which have possible scores of 0, 2, or 4. Total scores of 1 to 7 are considered low-risk according to the World Health Organisation guidelines, scores of 8 to 14 suggest hazardous alcohol consumption, and scores of 15 or more indicate the likelihood of alcohol dependence.

The 60-item short version of the HEXACO questionnaire was used to assess personality across six dimensions, Honesty-Humility, Emotionality, Extraversion, Agreeableness, and Openness, with 10 questions per dimension [29]. Items are rated on a 5-point scale (1 = ‘strongly disagree’, 5 = ‘strongly agree’). The HEXECO 60-item version demonstrates high internal consistency (α = 0.73–α = 0.80).

The 20-item Attentional Control Scale [30] was used to assess attentional control. Participants are presented with a series of statements and asked to rate how frequently each applies to them (1 = Almost Never, 4 = Always). The scale has a high internal consistency (α = 0.88).

Emotional dysregulation was assessed via the 36-item Difficulties in Emotion Regulation Scale (DERS) [31]. This scale has a high internal consistency (α = 0.93) and asks participants to rate how frequently each statement applies to them (1 = Almost Never, 5 = Almost Always). The scale gives a total score and contains six factors; ‘Non-Acceptance’, ‘Goals’, ‘Impulse Control’, ‘Awareness’, ‘Strategies’, and ‘Clarity’.

### 2.4. Statistical Analysis

Data were collected via Qualtrics and analysed using SPSS (version 28). Scores were calculated as per instructions for each scale. Estimated Blood Alcohol Concentration (eBAC) was calculated via the Widmark formula [32]. Three models were analysed using multiple linear regression. The first model tested whether hangover severity (mean mAHSS scores) was predicted by personality, attentional control, emotion dysregulation, alcohol consumption (eBAC), sex, or age. The second model tested whether the impact of a hangover on daily tasks was predicted by the above factors and hangover severity, and the third model tested whether AUDIT scores were predicted by the above factors and hangover severity. Cook’s Distance was used to identify influential cases and cases that were 3× the mean of all distances were removed (nine in model one, eight in model two, and five in model three). Following the removal of influential cases, models were re-run and each model met the assumptions for multiple linear regression. Results were considered significant if *p* < 0.05.

## 3. Results

### 3.1. Alcohol Consumption

On average, participants consumed 11.47 units of alcohol (*sd* = 7.32) during their last drinking episode that resulted in a hangover, lasting for an average of 6.5 h (*sd* = 2.5) and reaching a peak eBAC of 0.11%.

### 3.2. Hangover Severity

Hangover severity scores indicated that participants last-experienced hangover was mild-to-moderate (*M* = 4.22, *SD* = 1.75). Whilst controlling for eBAC, total attentional control (*r* = −0.27, *p* = 0.008) and total emotion dysregulation (*r* = 0.53, *p* < 0.001) scores significantly correlated with hangover severity. Table 1, Table 2 and Table 3 presents a correlation coefficient for each sub-factor.

The overall regression was statistically significant (Adj. R^2^ = 0.46, *F*(17,81) = 5.807, *p* < 0.001), with Focusing (*B* = −0.096, *p* = 0.011, 95%CI = −0.169–−0.023) and Agreeableness (*B* = 0.072, *p* = 0.005, 95%CI = 0.022–0.123) significantly predicting hangover severity (Table 1).

### 3.3. Impact

In the current study, participants reported that the impact of their last-experienced hangover was 9.77 (*SD* = 4.95). For reference, previous surveys for a community sample with some form of self-reported illness reported an average score of 7.96 (*SD* = 6.36), and a sample of psychiatric patients reported scores of 15.66 (*SD* = 4.04) [27]. Whilst controlling for eBAC, total attentional control (*r* = −0.27, *p* = 0.007) and total emotion dysregulation (*r* = 0.45, *p* < 0.001) scores significantly correlated with the impact of a hangover.

All predictors included in the model for hangover severity were included in this model, with the additional predictor of hangover severity (mAHSS scores). The overall regression was statistically significant (Adj. R^2^ = 0.41, *F*(18, 81) = 4.800, *p* < 0.001), with Shifting (*B* = −0.252, *p* = 0.021, 95%CI = −0.466–−0.038), Extraversion (*B* = 0.225, *p* = 0.009, 95%CI = 0.059–0.391), Agreeableness (*B* = −0.156, *p* = 0.042, 95%CI = −0.306–−0.005), and Hangover Severity (*B* = 1.603, *p* < 0.001, 95%CI = 1.020–2.187) significantly predicting the impact of a hangover on daily activities (Table 2).

### 3.4. AUDIT Scores

Total AUDIT scores indicated that, on average, participants were within the ‘increasing risk’ category (*M* = 10.58, *SD* = 5.06). Whilst controlling for eBAC, total emotion dysregulation (*r* = 0.35, *p* < 0.001), but not total attentional control (*r* = −0.05, *p* = 0.644) scores significantly correlated with AUDIT scores.

**Figure 1:**
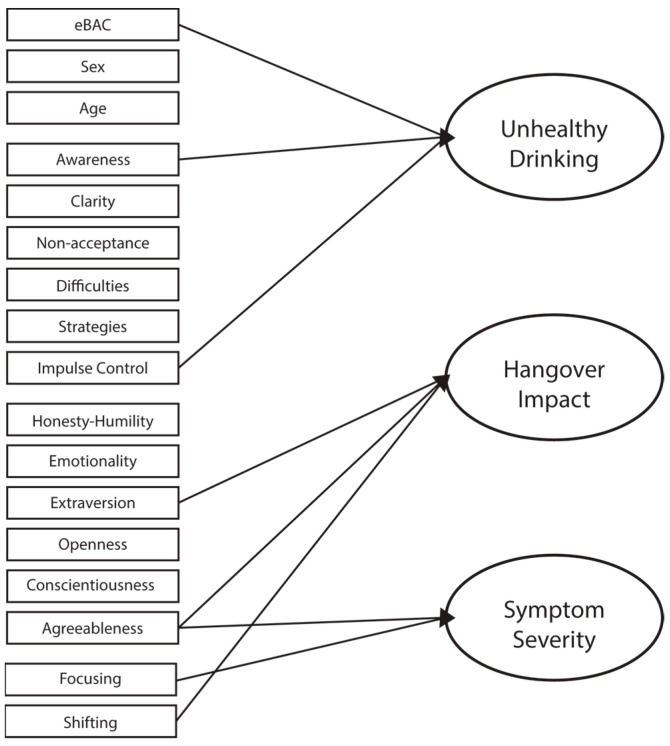
A visual representation of the link between unique predictors and each outcome variable. Arrows between predictors and outcomes are significant at *p* < 0.05.

All predictors were included in this model, including hangover severity (mAHSS scores) and impact (IIS scores). The overall regression was statistically significant (Adj. R^2^ = 0.45, *F*(19,83) = 5.318, *p* < 0.001), with the emotion dysregulation factors ‘Awareness’ (*B* = 0.301, *p* = 0.044, 95% CI = 0.008–0.594) and ‘Impulse control’ (*B* = 0.381, *p* = 0.011, 95% CI = 0.088–0.673), and eBAC (*B* = 25.051, *p* = 0.001, 95% CI = 11.279–38.822) significantly predicting AUDIT scores (Table 3). A visual diagram of results is presented in Figure 1.

## 4. Discussion

The current study aimed to explore how personality and factors related to self-regulation contribute toward the experience of a hangover, its impact on daily activities, and unhealthy drinking behaviours. In line with our hypothesis, each multiple linear regression with personality, attentional control, and emotion dysregulation included as predictors of hangover severity, hangover impact, and unhealthy drinking behaviours were significant. Each model also suggests factors that uniquely predict the criterion variable. The attentional control factor ‘focusing’ predicted lower hangover severity, whilst the factor ‘shifting’ predicted lower impact. Greater difficulties in awareness of emotions predicted greater AUDIT scores. However, counter to our hypothesis, regulatory factors did not uniquely predict hangover severity. Extraversion predicted greater impact of hangover, but did not uniquely predict hangover severity or AUDIT scores.

Previous research has been mixed regarding the link between hangover and personality, with some suggesting neuroticism predicts hangover experience [6], and others finding no association between personality and severity [7]. Our findings provide additional evidence for the link between personality and severity with results indicating that agreeableness positively predicts severity. However, other personality factors, including extraversion did not uniquely predict severity scores. Furthermore, those scoring higher in agreeableness indicated a lower impact of hangover on daily activities whereas higher scores of extroversion indicated a greater impact. Extroverted individuals tend to cope by seeking social support [12], and qualitative studies suggest benefits of communal bonding during hangover [11]. Therefore, one explanation of the link between extraversion and a greater impact of hangover is that extroverted individuals spend time reminiscing and coming to terms with their drinking activities and hangover experience in an attempt to alleviate their negative experience at the expensive of completing daily chores. This may be in direct contrast to those high on agreeableness, where individuals may be more willing to compromise their own needs to continue completing their daily activities, particularly as previous research indicates agreeableness is positively correlated with problem-focused coping styles [33]. Contrary to previous research [8], our results did not reveal any personality factor as a unique predictor of unhealthy drinking when the statistical model included regulatory and attentional control factors. Nor did our results replicate findings suggesting a link between hangover severity and future problematic drinking [21]. Our results did highlight the unique predictive validity of regulatory strategies and unhealthy drinking. Specifically, difficulties in impulse control and emotional awareness predicted AUDIT scores. However, eBAC was by far the biggest predictor of unhealthy drinking, highlighting that the link between hangover and future problematic drinking may be driven by alcohol consumption and regulatory processes, rather than mechanisms unique to hangover experience.

Our results highlight that attentional control is negatively related to hangover severity and the impact of a hangover. They also expand on our understanding of the role attention plays in the hangover experience by highlighting that the attentional control processes underlying subjective experience and the ability to continue with daily activities differ. For severity, lower levels of the ability to focus attention and inhibit distracting information predicted greater subjective hangover severity. These results imply that the reduction in attentional resources available for completing tasks during a hangover is, at least partially, influenced by the symptoms experienced during a hangover. Painful symptoms of a hangover, such as headache, consume attention [17] and so individuals who score lower in their ability to maintain focus may have greater interference from these attentionally consuming symptoms resulting in higher subjective severity scores. Interestingly, the impact of a hangover on daily activities is predicted by the attentional control factor of ‘shifting’, i.e., the ability to switch our attention between multiple tasks. Previous research highlights hangover-related impairments in the ability to multi-task [34] and switch attention [13]. Reduced available attentional resource to dedicate to tasks, alongside these cognitive impairments may underlie difficulties in one’s ability to complete daily activities, such as household chores, particularly for those who score lower in attentional shifting. However, the link between attentional capacity and task completion during hangover is still to be established.

Although our results established a positive association between total difficulties in emotion regulation and hangover severity, and a multiple regression model that included each subfactor of the DERS significantly predicted severity, no subfactor uniquely predicted severity. This is surprising, particularly given that previous research has indicated difficulties in modulation and clarity positively correlate with severity [13]. Our model also indicated that difficulties in impulse control and the awareness of emotions predicted unhealthy drinking. This is in line with previous research suggesting the importance of emotional dysregulation in the development and maintenance of alcohol use disorders [23,35,36].

The current study is not without limitations that should be acknowledged when interpreting results. The study did not measure, and therefore statistically control for, subjective intoxication and the frequency with which individuals experience hangovers. These have recently been highlighted as important factors, both in terms of the hangover experience and its potential role in future problematic drinking and other alcohol-related harms [9,37,38,39]. The study also did not measure physiological factors that are known to be associated with the severity of a hangover, such as cytokines [40]. These were beyond the scope of the current study. Although age was included within the model due to recent findings suggesting hangover severity declines with age [41], the mean age of the current sample was relatively young (23-years-old) and so may not have been sufficiently heterogeneous to uncover potential relationships. Furthermore, other demographic factors that are influential for personality, such as income and ethnicity, were not included. However, a key strength of the current study is the use of multiple linear regression, as opposed to multiple bivariate correlations, which are more typical in hangover studies, e.g., ref. [7]. Multiple linear regression allows a researcher to statistically control for shared variance between predictors and uncover factors that have a unique contribution toward predicting the outcome variable. This reduces the chance of associations being made that are driven by underlying processes common with other factors. For example, the ability to regulate emotions requires the use of attentional control, both of which correlated with hangover severity in our study. However, when subfactors were included in the regression model that accounts for shared variance, only ‘Focusing’ attentional control uniquely predicted hangover severity. Caution should be taken when interpreting the magnitude of these effects though, as each predictor identified had small *sr*^2^ values—the amount of variance that is uniquely predicted by the factor.

## 5. Conclusions

Overall, results from this study highlight that attentional control, emotion regulation, and personality predict hangover severity. Although emotion regulation is a key process to overcome adverse situations, our results suggest that the underlying functions of attentional control may be driving associations between regulation and hangover. Furthermore, our results indicate that participants scoring high in agreeableness tended to have more severe hangovers but less impact on their daily activities. In addition, personality, attentional control, and emotion regulation predicted unhealthy drinking behaviours, with difficulties in impulse control, emotional awareness, and eBAC as unique predictors.

## Figures and Tables

**Table 1:** Regression results using mean hangover severity scores as the criterion.

Predictor	*b*	*b* 95% CI [LL, UL]	*beta*	*beta* 95% CI [LL, UL]	*sr* ^2^	*sr*^2^ 95% CI [LL, UL]	*r*	Fit
(Intercept)	3.25	[−2.04, 8.54]						
Focusing	−0.10 *	[−0.17, −0.02]	−0.25	[−0.44, −0.06]	0.04	[−0.01, 0.09]	−0.39 **	
Shifting	0.03	[−0.04, 0.10]	0.09	[−0.11, 0.28]	0.00	[−0.01, 0.02]	−0.27 **	
Honesty	−0.05	[−0.10, 0.00]	−0.20	[−0.40, 0.01]	0.02	[−0.02, 0.06]	−0.26 **	
Emotionality	0.01	[−0.04, 0.06]	0.02	[−0.19, 0.24]	0.00	[−0.00, 0.00]	0.21 *	
Extraversion	−0.05	[−0.10, 0.00]	−0.20	[−0.41, 0.01]	0.02	[−0.02, 0.06]	−0.36 **	
Agreeableness	0.07 **	[0.02, 0.12]	0.26	[0.08, 0.44]	0.05	[−0.01, 0.10]	0.03	
Conscientiousness	−0.00	[−0.05, 0.04]	−0.01	[−0.20, 0.17]	0.00	[−0.00, 0.00]	−0.23 *	
Openness	−0.02	[−0.06, 0.02]	−0.08	[−0.24, 0.08]	0.01	[−0.01, 0.02]	−0.10	
Nonacceptance	0.04	[−0.02, 0.10]	0.14	[−0.08, 0.37]	0.01	[−0.02, 0.03]	0.42 **	
Difficulties	0.01	[−0.07, 0.10]	0.03	[−0.19, 0.26]	0.00	[−0.01, 0.01]	0.39 **	
Awareness	−0.00	[−0.08, 0.08]	−0.01	[−0.22, 0.20]	0.00	[−0.00, 0.00]	0.01	
Clarity	0.07	[−0.01, 0.16]	0.20	[−0.03, 0.43]	0.02	[−0.02, 0.05]	0.53 **	
Strategies	0.00	[−0.07, 0.08]	0.01	[−0.30, 0.32]	0.00	[−0.00, 0.00]	0.48 **	
Impulse	0.06	[−0.02, 0.13]	0.17	[−0.06, 0.40]	0.01	[−0.02, 0.04]	0.49 **	
eBAC	1.95	[−1.77, 5.67]	0.10	[−0.09, 0.29]	0.01	[−0.01, 0.03]	0.15	
Sex	0.63	[−0.14, 1.39]	0.17	[−0.04, 0.38]	0.01	[−0.02, 0.05]	0.22 *	
Age	−0.00	[−0.05, 0.04]	−0.02	[−0.20, 0.16]	0.00	[−0.00, 0.00]	−0.29 **	
								*R*^2^ = 0.549 **
								95% CI [0.29, 0.58]

Note. A significant *b*-weight indicates the beta-weight and semi-partial correlation are also significant. *b* represents unstandardized regression weights. *beta* indicates the standardized regression weights. *sr*^2^ represents the semi-partial correlation squared. *r* represents the zero-order correlation. *LL* and *UL* indicate the lower and upper limits of a confidence interval, respectively. * indicates *p* < 0.05. ** indicates *p* < 0.01.

**Table 2:** Regression results using total impact of hangover scores as the criterion.

Predictor	*b*	*b* 95% CI [LL, UL]	*beta*	*beta* 95% CI [LL, UL]	*sr* ^2^	*sr*^2^ 95% CI [LL, UL]	*r*	Fit
(Intercept)	7.80	[−8.11, 23.70]						
Focusing	0.07	[−0.16, 0.31]	0.07	[−0.14, 0.27]	0.00	[−0.01, 0.02]	−0.26 **	
Shifting	−0.25 *	[−0.47, −0.04]	−0.25	[−0.46, −0.04]	0.03	[−0.02, 0.08]	−0.30 **	
Honesty	0.13	[−0.03, 0.29]	0.17	[−0.04, 0.38]	0.02	[−0.02, 0.05]	−0.17	
Emotionality	−0.02	[−0.16, 0.12]	−0.02	[−0.23, 0.18]	0.00	[−0.00, 0.01]	0.13	
Extraversion	0.22 **	[0.06, 0.39]	0.29	[0.08, 0.50]	0.04	[−0.01, 0.10]	−0.10	
Agreeableness	−0.16 *	[−0.31, −0.01]	−0.19	[−0.38, −0.01]	0.03	[−0.02, 0.07]	−0.21 *	
Conscientiousness	−0.01	[−0.15, 0.12]	−0.02	[−0.20, 0.17]	0.00	[−0.00, 0.00]	−0.24 *	
Openness	−0.08	[−0.20, 0.04]	−0.11	[−0.28, 0.06]	0.01	[−0.02, 0.04]	−0.12	
Nonacceptance	0.08	[−0.11, 0.27]	0.10	[−0.13, 0.34]	0.00	[−0.01, 0.02]	0.38 **	
Difficulties	−0.12	[−0.39, 0.15]	−0.10	[−0.33, 0.13]	0.00	[−0.01, 0.02]	0.31 **	
Awareness	−0.25	[−0.50, 0.00]	−0.21	[−0.43, 0.00]	0.02	[−0.02, 0.06]	−0.01	
Clarity	0.15	[−0.14, 0.44]	0.13	[−0.12, 0.38]	0.01	[−0.02, 0.03]	0.35 **	
Strategies	0.21	[−0.03, 0.44]	0.29	[−0.04, 0.62]	0.02	[−0.02, 0.06]	0.42 **	
Impulse	−0.13	[−0.38, 0.13]	−0.12	[−0.38, 0.13]	0.01	[−0.01, 0.03]	0.29 **	
eBAC	−0.04	[−6.84, 6.77]	−0.00	[−0.17, 0.17]	0.00	[−0.00, 0.00]	0.05	
Sex	−1.17	[−3.33, 1.00]	−0.11	[−0.31, 0.09]	0.01	[−0.02, 0.03]	0.10	
Age	0.01	[−0.11, 0.13]	0.01	[−0.17, 0.20]	0.00	[−0.00, 0.00]	−0.16	
Severity	1.60 **	[1.02, 2.19]	0.56	[0.35, 0.76]	0.18	[0.07, 0.29]	0.60 **	
								*R*^2^ = 0.516 **
								95% CI [0.24, 0.54]

Note. A significant *b*-weight indicates the beta-weight and semi-partial correlation are also significant. *b* represents unstandardized regression weights. *beta* indicates the standardized regression weights. *sr*^2^ represents the semi-partial correlation squared. *r* represents the zero-order correlation. *LL* and *UL* indicate the lower and upper limits of a confidence interval, respectively. * indicates *p* < 0.05. ** indicates *p* < 0.01.

**Table 3:** Regression results using total AUDIT scores as the criterion.

Predictor	*b*	*b* 95% CI [LL, UL]	*beta*	*beta* 95% CI [LL, UL]	*sr* ^2^	*sr*^2^ 95% CI [LL, UL]	*r*	Fit
(Intercept)	0.92	[−17.86, 19.70]						
Focusing	0.02	[−0.25, 0.30]	0.02	[−0.18, 0.21]	0.00	[−0.00, 0.00]	−0.15	
Shifting	−0.02	[−0.28, 0.24]	−0.02	[−0.22, 0.19]	0.00	[−0.00, 0.00]	−0.13	
Honesty	−0.12	[−0.31, 0.06]	−0.14	[−0.34, 0.07]	0.01	[−0.02, 0.03]	−0.43 **	
Emotionality	−0.04	[−0.20, 0.13]	−0.04	[−0.24, 0.16]	0.00	[−0.01, 0.01]	−0.14	
Extraversion	0.19	[−0.01, 0.40]	0.21	[−0.01, 0.42]	0.02	[−0.02, 0.06]	0.12	
Agreeableness	0.13	[−0.05, 0.31]	0.13	[−0.05, 0.32]	0.01	[−0.02, 0.04]	−0.01	
Conscientiousness	−0.06	[−0.23, 0.10]	−0.07	[−0.24, 0.11]	0.00	[−0.01, 0.02]	−0.27 **	
Openness	−0.07	[−0.21, 0.06]	−0.09	[−0.25, 0.07]	0.01	[−0.01, 0.03]	−0.10	
Non-acceptance	−0.05	[−0.27, 0.16]	−0.05	[−0.27, 0.17]	0.00	[−0.01, 0.01]	0.26 **	
Difficulties	−0.00	[−0.31, 0.31]	−0.00	[−0.22, 0.22]	0.00	[−0.00, 0.00]	0.13	
Awareness	0.30 *	[0.01, 0.59]	0.22	[0.01, 0.43]	0.02	[−0.02, 0.06]	0.13	
Clarity	−0.05	[−0.37, 0.28]	−0.03	[−0.27, 0.20]	0.00	[−0.00, 0.01]	0.29 **	
Strategies	0.04	[−0.24, 0.32]	0.04	[−0.27, 0.36]	0.00	[−0.00, 0.01]	0.17	
Impulse	0.38 *	[0.09, 0.67]	0.31	[0.07, 0.54]	0.04	[−0.01, 0.09]	0.33 **	
eBAC	25.05 **	[11.28, 38.82]	0.35	[0.16, 0.54]	0.07	[0.00, 0.14]	0.48 **	
Sex	−1.55	[−4.23, 1.13]	−0.11	[−0.31, 0.08]	0.01	[−0.01, 0.03]	−0.09	
Age	−0.11	[−0.25, 0.04]	−0.14	[−0.32, 0.04]	0.01	[−0.02, 0.04]	−0.32 **	
Hangover Severity	0.56	[−0.22, 1.35]	0.16	[−0.06, 0.39]	0.01	[−0.02, 0.04]	0.41 **	
Hangover Impact	0.13	[−0.11, 0.36]	0.11	[−0.09, 0.31]	0.01	[−0.01, 0.03]	0.31 **	
								*R*^2^ = 0.549 **
								95% CI [0.28, 0.57]

Note. A significant *b*-weight indicates the beta-weight and semi-partial correlation are also significant. *b* represents unstandardized regression weights. *beta* indicates the standardized regression weights. *sr*^2^ represents the semi-partial correlation squared. *r* represents the zero-order correlation. *LL* and *UL* indicate the lower and upper limits of a confidence interval, respectively. * indicates *p* < 0.05. ** indicates *p* < 0.01.

## Data Availability

The data presented in this study are available on request from the corresponding author.
